# Social Factors in Aesthetics: Social Conformity Pressure and a Sense of Being Watched Affect Aesthetic Judgments

**DOI:** 10.1177/2041669517736322

**Published:** 2017-11-16

**Authors:** Vera M. Hesslinger, Claus-Christian Carbon, Heiko Hecht

**Affiliations:** Abteilung Allgemeine Experimentelle Psychologie, University of Mainz, Germany; Department of General Psychology and Methodology, University of Bamberg, Germany; Forschungsgruppe EPÆG (Ergonomics, Psychological Æsthetics, Gestalt), Germany; Department of General Psychology and Methodology, University of Bamberg, Germany; Forschungsgruppe EPÆG (Ergonomics, Psychological Æsthetics, Gestalt), Germany; Abteilung Allgemeine Experimentelle Psychologie, University of Mainz, Germany

**Keywords:** eyespots, aesthetic judgments, conformity, social factors, empirical aesthetics

## Abstract

The present study is a first attempt to experimentally test the impact of two specific social factors, namely social conformity pressure and a sense of being watched, on participants’ judgments of the artistic quality of aesthetic objects. We manipulated conformity pressure with a test form in which a photograph of each stimulus was presented together with unanimously low (downward pressure) or high quality ratings (upward pressure) of three would-be previous raters. Participants’ sense of being watched was manipulated by testing each of them in two settings, one of which contained an eyespots stimulus. Both social factors significantly affected the participants’ judgments—unexpectedly, however, with conformity pressure only working in the downward direction and eyespots leading to an overall downward shift in participants’ judgments. Our findings indicate the relevance of including explicit and implicit social factors in aesthetics research, thus also reminding us of the limitations of overly reductionist approaches to investigating aesthetic perception and experience.

*I thought to discourage aesthetics … I threw the bottle rack and the urinal in their faces and now they admire them …*

Marcel Duchamp

When strolling through a museum, we see artworks and we somehow react to what we are seeing, emotionally and cognitively (e.g., Kreitler, & Kreitler, 1972). We form opinions and we judge the artworks, for instance, with regard to aesthetic appeal, interestingness, or artistic quality. As empirical aesthetics research has shown, such aesthetic judgments are not determined by properties of the artworks alone, but also by a variety of external factors (i.e., factors that are not actual properties of the artworks). Mere exposure, for instance, is capable of increasing recipients’ preferences for artworks, and even of promoting whole artistic canons (Cutting, 2003). Aesthetic judgments are further affected or modulated by semantic and physical context (Locher, Smith, & Smith, 2001; Muth, Raab, & Carbon, 2017), training and experience (e.g., Nodine, Locher, & Krupinski, 1993), as well as expertise (e.g., Pihko et al., 2011), to name just a few. Besides, judgments of artistic quality are relatively easy to manipulate by providing information about an artwork’s authenticity (Wolz & Carbon, 2014). Even the perceived artistic quality of an acclaimed masterpiece such as Leonardo’s *La Gioconda* can be sabotaged, simply by making recipients deeply elaborate an alternative version of the painting (the rediscovered and restored “Prado version,” presented to the public in 2012; see Carbon & Hesslinger, 2013) for just 10 minutes (Carbon & Hesslinger, 2014).

According to several theoretical accounts, social variables are being incorporated into aesthetic reception and the formation of aesthetic judgments (e.g., Muelder Eaton, 1995; Mukařovský, 2015; Riemer, Shavitt, Koo, & Markus, 2014). Empirical support for this notion stems from observations in museums. For instance, Tröndle, Wintzerith, Wäspe, and Tschacher (2012) found that companionship and conversation of museum visitors affected their reception of the exhibited artworks. In a recent field study carried out by Carbon (2017), these two types of social behavior were shown by many of the observed museum visitors. However, empirical and especially experimental inquiries into the role of different social variables with regards to aesthetic reception and judgments are sparse. Madden (1960) made one of the rare experimental attempts and tested whether judgments of facial beauty could be modified by exertion of social conformity pressure. The paradigm used in the experiment was a modified version of the paradigm used by Asch (1956). In a now classic series of experiments, Asch investigated how strongly judgments in a simple perceptual comparison task conform to or are independent of the judgments of a unanimous majority. Participants were tested in groups of 8 to 10 men; they had to compare a standard line against three comparison lines, one of which most obviously had the same length as the standard line. In the most basic variant of the paradigm, judgments had to be stated aloud in front of the group. All participants but one were in fact confederates of the experimenter and had been instructed beforehand to give unanimously wrong answers in certain trials. Thus, the only “real” tested individual, who always was the second-to-last to announce his judgment, faced conformity pressure directed counter the correct answer in these trials. Madden (1960) modified the Asch paradigm in that the group exerting social conformity pressure was not physically present in the experimental setting; instead, this social background was simulated by presenting ratings that were labeled as “given by previous raters.” Each participant was asked to judge several profile drawings of female heads with regards to beauty on a scale from 1 (*homely*) to 7 (*beautiful*), and to indicate his judgments by writing them onto a rating form which had been prepared by the experimenter to look as if it had already been used by some previous participants. The number of these simulated or made-up participants was either one (for two subsamples), three (for eight subsamples), or five (for two subsamples). On 15 critical trials, the simulated ratings were either two scale points higher than the average that the respective stimulus had received in a pre-study (upward conformity pressure, exerted for one half of the subsamples) or two scale points lower (downward conformity pressure, exerted for the other half of the subsamples). The results indicate that the experimentally induced social conformity pressure swayed the participants’ beauty judgments toward the manipulated average, working especially well in the downward direction.

Besides judgments of facial beauty, music preferences of adolescents (Inglefield, 1968) and judgments of present and future fashionability of clothing (Davis & Miller, 1983) were shown to be affected by social conformity pressure in experimental settings. With the present experiment, we transpose the research on the impact of social conformity pressure to another domain of aesthetics, the reception and judgment of aesthetic objects, or more specifically to the judgment of their artistic quality. We hypothesize that similar to judgments of beauty, judgments of artistic quality are likewise influenced by social conformity pressure. To test this, we adopted Madden’s (1960) paradigm of exerting social conformity pressure on our participants while they were making judgments of artistic quality. This allowed us to conduct a modified replication of Madden’s study. Moreover, it allowed us to use perceived artistic quality as a dependent measure, thus going beyond mere ratings of preference or impressions of beauty. Although related to preference, artistic quality goes beyond, as it is a multidimensional construct, determined by, inter alia, originality and ambiguity (see Haertel & Carbon, 2014). As stimuli, we used photographs of objects with indifferent (i.e., medium) artistic quality, which had been assessed in an earlier baseline study. This is in line with Madden, who showed female profiles of, previously rated, medium beauty. We hypothesized that participants would reorient their judgments in the direction of the social conformity pressure.

As past research has shown that normative behavior can be increased by observation (Munger & Harris, 1989), we exerted conformity pressure under two different conditions. Either we merely displayed manipulated ratings of would-be previous participants, or we added a photograph of a pair of eyes to these fake ratings. Images of eyes or, more generally, eyespots stimuli can serve as cues eliciting a sense of being seen or watched (Pfattheicher & Keller, 2015). The mere presence of eyespots was found to be effective in enhancing various kinds of pro-social behavior in the laboratory as well as in real-world settings (e.g., Bateson, Nettle, & Roberts, 2006; Haley & Fessler, 2005). In contrast to the confrontation with fake ratings of additional would-be raters, which represents a quite explicit cue to the social embedding of the participants’ judgments, eyespots stimuli are considered as rather implicit social cues or triggers (see, e.g., Panagopoulos & van der Linden, 2017). Including an eyespots-present condition in the present experiment, we thus introduced a second, qualitatively different source of social influence. We accordingly hypothesized that the conformity pressure would be further increased by the presence of eyespots so that the difference between judgments made under downward versus upward conformity pressure would be higher in the eyespots-present setting than in the eyespots-absent setting.

It has been theorized and shown that aesthetic judgments may be moderated by person-related variables such as art expertise (e.g., Kreitler, & Kreitler, 1972; Pihko et al., 2011). Responsiveness to social cues and pressures, on the other hand, may vary due to individual differences in certain personality factors. DeYoung, Peterson, and Higgins (2002), for instance, assessed conformity in terms of socially desirable responding and found conformity to be positively related with stability, but negatively with plasticity, both of which are, according to them, higher-order factors of the Big Five. In order to be able to test, in a secondary analysis, whether results obtained in the present experiment were also affected by individual differences in the named variables (art expertise, social desirability, stability, and plasticity) we decided to measure them by means of questionnaires.

## Method

### Participants

A total of 48 students of the University of Bamberg (13 men; age *M* = 23.0 years, range: 18–34 years) took part in the experiment. They all showed normal or corrected-to-normal visual acuity and normal color vision. The participants were treated in accordance with the declaration of Helsinki and gave written informed consent before the experiment. They received a monetary compensation for their participation (€5 each).

### Design

The present experiment followed a 2 × 2 repeated measures design testing the impact of the factors *conformity pressure* (upward, downward) and *eyespots* (present, absent) on the perceived artistic quality of aesthetic stimuli. The design was fully crossed. Please note that, for the factor conformity pressure, we followed the approach of Madden (1960) and referred to previously collected baseline data on the artistic quality of the stimuli (Haertel & Carbon, 2014) instead of including a control group. The sample used for assessing the baseline data stemmed from the same participant pool as the sample used in the present experiment; both samples exclusively comprised students of the University of Bamberg with a comparable age composition (baseline sample: *N* = 17, age *M* = 24.1 years, range: 19–33 years, our sample: see section above). The levels of the factor *eyespots* were implemented in a blockwise way with the order of levels being counterbalanced across participants. The levels of the factor *conformity pressure* were varied within the blocks with eyespots being present and absent, respectively. Each block comprised, in pseudo-randomized order, five trials with upward and five trials with downward conformity pressure being exerted.

### Apparatus

#### Aesthetic stimuli

As aesthetic stimuli, we used color photographs of 20 different objects, 13 of which were contemporary art objects, and 7 of which were everyday objects. A brief description of each stimulus and, in the case of art objects, artist, title, and year of production are listed in Table 1. The 20 stimuli were chosen out of a larger set of photographed objects such as to avoid extreme average artistic quality ratings that might suffer from ceiling effects. Thus, we chose stimuli that had received a medium rating with regards to this variable in a previous study by Haertel and Carbon (2014), more precisely an average rating for artistic quality of minimally 3 and maximally 5 on a scale ranging from 1 (*very low artistic quality*) to 7 (*very high artistic quality*). The 20 selected stimuli were grouped into two parallelized subsets (Set A, Set B) of 10 stimuli each. To ensure comparable levels of artistic quality in both subsets, this was done by generating 10 pairs of stimuli with matching baseline ratings for artistic quality (Table 2). Mean artistic quality ratings of the two subsets did not differ significantly, *t*(9) < 1, *p* = .3722, *ns*.

**Table 1.** Brief Descriptions of the Aesthetic Stimuli Used in the Present Experiment.

Label	Description of Stimulus.
A01	Elegant red-orange gemmed stilettos arranged with red-black lace lingerie on grayish background
A02	Bottom of a painted wooden skateboard without wheels photographed against a white background, painted blue-red motif showing a cat standing on scissors cutting an apple (Jim Avignon, “I am your PM,” date unknown)
A03	Little tower made of 12 rows of multicolored textile bricks with one black shoe standing on top, installed on a tiled floor (Michelangelo Pistoletto, “Little monument,” 1968)
A04	Black conductor’s bag with coin dispenser and brass clasp, three “DKV” (Deutsche Krankenversicherung) stickers attached to the bag
A05	Assemblage of a black metal (or plastic) corpus, several medical capsules, yellow plastic tubes of different lengths, and pieces of dark pink napkin, installed against two horizontal fields colored yellow and turquoise (Samuel Henne, part of the work “Something specific about everything,” 2010/2011)
A06	Artificial rose made of magazine paper in glass bottle against black background (Sarah Illenberger, “Magazine flower,” probably 2005)
A07	Assemblage of artificial fruits made of ceramics, installed against white background (Christoph Schellberg, “Crumble,” 2010)
A08	Circular installation of white shoes on dark tiled floor (Mandy Hilse, “Lebenslauf,” 2008)
A09	Wooden cutting board with lying salt cellar against white-grayish background
A10	Blue igloo-shaped tent installed inside a room; names appliqued at the insides of the tent, dates (1963–1995) at the base of one of the outsides (Tracy Emin, “Everyone I have ever slept with 1963–1995”/“The tent,” 1995)
B01	Cracked green wine bottle shown against white background (Nicolas Boulard, “Fragments,” 2007)
B02	Playing card cut into pieces lying on wooden floor
B03	Assemblage of sewn and stuffed textile animals, felt, and a small basket (Mike Kelley, “Frankenstein,” 1989)
B04	Assemblage of multiple artifacts of various materials standing on wooden floor
B05	Close-up of a peeled pomegranate
B06	Bottom of pink orange skateboard with three holes cut into it (Boris Hoppek, title unknown, date unknown)
B07	Colorful piece of knitting put around a branch of a mossy tree
B08	Cut piece of cauliflower lying on surface with geometric pattern (Sarah Illenberger, “Blumenkohl,” date unknown)
B09	Glow-lamp in front of wooden surface, bulb replaced by a light green pear (Sarah Illenberger, “Birne,” date unknown)
B10	Vertical assemblage of wooden parts and rope against light grey background (Paul Joosten, title unknown, 1918)

*Note.* In the case of artworks, respective artist, title, and year have been mentioned.

**Table 2.** Baseline and “Fake” Artistic Quality Ratings for the Aesthetic Stimuli Used in the Present Experiment.

	Baseline ratings			Fake ratings upward				Fake ratings downward			
Match pair	Set A	Set B	*M*_base_	FR1	FR2	FR3	*M*_fake+_	FR1	FR2	FR3	*M*_fake−_
1	3.86	4.43	4.14	6.00	6.00	6.00	6.00	2.00	2.00	2.00	2.00
2	4.57	4.57	4.57	7.00	6.00	7.00	6.67	3.00	2.00	3.00	2.67
3	5.14	5.29	5.21	8.00	7.00	7.00	7.33	4.00	3.00	3.00	3.33
4	5.43	5.43	5.43	8.00	8.00	6.00	7.33	4.00	3.00	3.00	3.33
5	5.57	5.57	5.57	8.00	7.00	8.00	7.67	4.00	3.00	4.00	3.67
6	5.71	5.86	5.79	7.00	8.00	8.00	7.67	3.00	4.00	4.00	3.67
7	5.86	5.86	5.86	8.00	8.00	8.00	8.00	4.00	4.00	4.00	4.00
8	6.00	6.00	6.00	9.00	7.00	8.00	8.00	4.00	4.00	4.00	4.00
9	6.29	6.29	6.29	8.00	8.00	9.00	8.33	4.00	4.00	5.00	4.33
10	6.43	6.43	6.43	7.00	9.00	9.00	8.33	4.00	4.00	5.00	4.33

*Note.* The left part of the table shows the baseline ratings for the aesthetic stimuli used in the present experiment (10 per set; each line in the columns “Set A” and “Set B,” respectively, represents the average baseline of one specific stimulus obtained in a previous study). The ratings have been obtained and published by Haertel and Carbon (2014). Please note that we have transformed the original ratings by multiplication with 9/7 as we did not use the 7-point scale of the earlier study but a 9-point scale for assessing artistic quality. The columns “Set A” and “Set B,” respectively, indicate the transformed values. The middle and the right part of the table show the fake ratings that were presented to the participants as ratings made by three other raters (made-up raters/“fake raters” FR1, FR2, and FR3)—but were in fact made up in order to exert an upward (middle part of table) or downward (right part of table) conformity pressure on the participants. For reasons of better comparability, both stimuli of a specific match pair were combined with the same fake ratings. The arithmetic mean of the baseline, and the upward and the downward fake ratings, respectively, for each match pair is indicated by the columns *M*_base_, *M*_fake+_, and *M*_fake−._

#### Setting used for manipulating presence of eyespots

**Figure 1.**
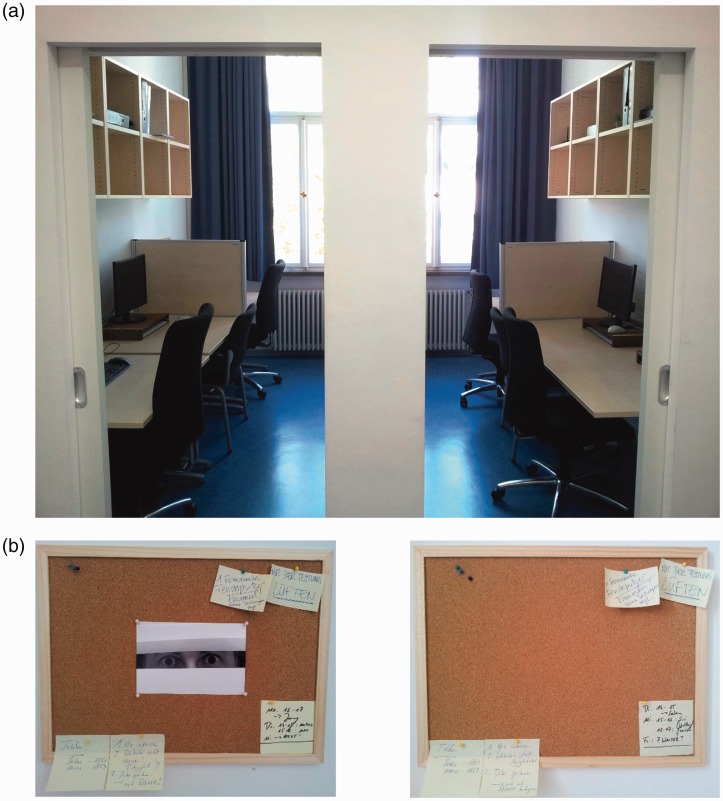
The test setting: View into the two test cabins (a), and photographs of the pin boards (b) that were fixed at the walls above the test places in the left (eyespots-present) and right cabin (eyespots-absent), respectively. *Source:* Photos taken by Vera M. Hesslinger.

The experiment was conducted in the Behavioral Lab at the University of Bamberg, which comprises an anteroom and two test cabins. The test cabins were furnished in a mirror-symmetric way with the permanent partition between the cabins being the axis of symmetry. Each cabin comprised a window vis-à-vis its entrance as well as two tables installed along the wall opposite the partition. The seat next to the window in the left and right cabin, respectively, was used for testing. Here, a pin board (29.5 cm × 41.5 cm) was fixed to the wall above the tabletop, roughly at the eye level of an average adult seated at the table. Several small notes (e.g., “Close window when leaving the room!”) were attached to the pin boards in both cabins to make the participants believe that these boards were not part of the experiment, but used for regular informal communication in the lab. The number of notes was the same in both cabins, but the content differed slightly. In the left cabin, a gray-scale close-up photography of a pair of male eyes staring through a shutter was further added to the pin board (*eyespots-present*). The photograph had a height of 110 mm and a width of 150 mm which is equal to the width of the watching-eyes images used by Bateson et al. (2006). In the right cabin, the respective space on the pin board was left blank (*eyespots-absent*). A view into the test cabins is depicted in Figure 1(a). Figure 1(b) shows the two pin boards.

#### Test form used for exerting conformity pressure

We created and used a test form in which the aesthetic stimuli were presented together with ratings of three would-be previous participants (cf. Madden, 1960). Our test form comprised an introductory instruction as well as two separate rating booklets containing one of the two parallelized subsets of aesthetic stimuli each. The introductory instruction told the participants that they were part of the pre-study of another experiment, which required gathering ratings for artworks from a large art database. The experimenter told each participant that they were the fourth out of a group of six raters judging a specific selection taken from this database. In each booklet, one of the 10 aesthetic stimuli of the respective subset was printed on an individual page together with six scales to indicate the artistic quality of the presented object. Each scale was reserved for one of the (would-be) raters in the actual participant’s group. In line with the cover story, in the first three scales, ratings had already been marked. This had been done by the first author beforehand, using a different pen and marks of a specific size for each of the three raters such that it looked as if different persons had already worked on the ratings. The actual participants were asked to use the next free (i.e., the fourth) scale to indicate their own judgment of artistic quality. The scale ranged from 1 = *very low artistic quality* to 9 = *very high artistic quality*. Our scale thus had a wider range than the 7-point scale previously used by Haertel and Carbon (2014) for gathering the baseline ratings. This allowed us to exert up- and downward conformity pressure on our participants’ judgments while keeping the risk of floor and ceiling effects low. Conformity pressure was exerted by shifting the averaged baseline ratings for each of the 10 match pairs of aesthetic stimuli by two categories on the 9-point scale. Concretely, we systematically selected the ratings of the made-up previous raters so that their average equaled about the averaged baseline ratings of the match pair in question plus 2 (*upward conformity pressure*) or minus 2 (*downward conformity pressure*). If, for example, upward conformity pressure should be exerted and the match pair had an average baseline of 4.6, the ratings of the would-be previous raters had to result in an arithmetic mean of about 6.6. Accordingly, we chose, for example, the values 7, 6, and 7 as ratings indicated by the made-up raters. For reasons of comparability, the same fake values were taken for both stimuli of a specific match pair. Baselines and fake ratings for the 10 match pairs of aesthetic stimuli are listed in Table 2. Up- and downward conformity pressure was induced for half of the 10 match pairs, respectively (i.e., for half of the stimuli in each rating booklet). To prevent confounding effects, we systematically varied the combination of specific stimuli with the direction of conformity pressure as well as the combination of stimulus set with setting (eyespots-present vs. eyespots-absent) over the sample. Table 3 gives a systematic overview of the different combinations that were used and the number of participants tested under each of these combinations. The sequence of the aesthetic stimuli in the rating booklets was pseudo-randomized (in sum, we had 32 different orders that were used for the sample).

**Table 3.** Systematic Overview of the Used Combinations of Setting, Stimuli, and Direction of Conformity Pressure.

Variant	Phase	Setting	Stimuli	Conformity pressure	*n*
1	T1	Eyespots	Set A	odd+/even−	7
	T2	No eyespots	Set B		
2	T1	Eyespots	Set A	odd−/even+	6
	T2	No eyespots	Set B		
3	T1	Eyespots	Set B	odd+/even−	5
	T2	No eyespots	Set A		
4	T1	Eyespots	Set B	odd−/even+	6
	T2	No eyespots	Set A		
5	T1	No eyespots	Set A	odd+/even−	5
	T2	Eyespots	Set B		
6	T1	No eyespots	Set A	odd−/even+	6
	T2	Eyespots	Set B		
7	T1	No eyespots	Set B	odd+/even−	7
	T2	Eyespots	Set A		
8	T1	No eyespots	Set B	odd−/even+	6
	T2	Eyespots	Set A		

T1 = first rating phase, participants worked through first rating booklet; T2 = second rating phase, participants worked through second rating booklet; Eyespots = rating took place in the left test cabin where eyespots were present; No eyespots = rating took place in the right test cabin where no eyespots were present; Set A = stimuli A01 to A10; Set B = stimuli B01 to B10; odd+/odd− = upward/downward conformity pressure was exerted for stimuli with odd stimulus numbers; even+/even− = upward/downward conformity pressure was exerted for stimuli with even stimulus numbers (please note that stimuli were presented in pseudo-randomized order, that is, numbering of the stimuli was not identical with order of presentation).

#### Questionnaires used for assessing person-related variables

To identify individuals with high or low expertise, respectively, we assessed our participants’ art expertise by means of several questions concerning their direct contact with the arts (e.g., “Wie oft haben Sie im letzten Jahr Kunstausstellungen besucht?/How often have you attended art exhibitions last year?”; “Wie viele Bücher über Kunst/Künstler besitzen Sie?/How many books about the arts or artists do you own?”).

In order to be able to control whether potential conformity effects with regards to judgments of artistic quality can also (at least partly) be traced back to social desirability, and whether it is further related to the two higher-order Big Five factors stability and plasticity, we assessed social desirability using a German version of the Brief Inventory of Desirable Responding/BIDR (Musch, Brockhaus, & Bröder, 2002) as well as the Big Five using a short German version of the Big Five Inventory/BFI-K (Rammstedt & John, 2005).

### Procedure

The present experiment comprised three main phases completed by each participant individually: an introductory phase and two test phases separated by a short break. The introductory phase and the first test phase were realized either in the left (*eyespots-present*, *n*_1_ = 24) or the right cabin (*eyespots-absent*, *n*_2_ = 24) of the lab, and the second test phase was conducted in the other cabin. During the break, which was introduced to temporally separate the test phases and to distract from the change of cabins, the participants were asked to come to the anteroom of the laboratory. In the introductory phase, participants read the instructions and received the monetary compensation for taking part in the experiment. The money was handed in a small pouch containing €5 partitioned into coins (1 × €2, 1 × €1, 2 × 50¢, 5 × 20¢, 10 × 10¢). If the participants had any questions concerning the procedure, these were answered by the experimenter. In the first test phase, participants worked through the first rating booklet judging the depicted aesthetic stimuli one after the other, in the given order without time constraints. During the break, the experimenter tested the participants’ visual abilities via a Snellen eye chart and an Ishihara color vision test (short version); furthermore, the participants were requested to fill in the personality and art expertise questionnaires. In the second test phase, they worked through the second rating booklet, just as they had done in the first test phase. At the end of the second rating booklet, after all ratings had been finished, they found an additional short note asking whether they would like to spend part of the monetary compensation they had received at the beginning of the experiment for a municipal social project (Lebenshilfe Bamberg). They were instructed to put the according amount of money (ranging from €0 to €5 in steps of 10¢) into an envelope lying on the table next to them and then throw the envelope into a black box so that the experimenter did not know whether or how much the individual participant had spent. We included the measure of donation behavior to enable a comparison with the results of previous studies that have shown an enhancing effect of eyespots on various pro-social behaviors such as donating (e.g., Keller & Pfattheicher, 2011). Except for the introductory phase and the break, the participants were left alone to prevent potential confounding effects related to the presence of the experimenter who might, despite any precautions, be perceived as watching or observing by the participants (concerning the role of real potential observers with regards to eyespots effects; see, e.g., Ekström, 2012). Participants took about 20 to 30 minutes to complete the entire experiment.

## Results

**Figure 2.**
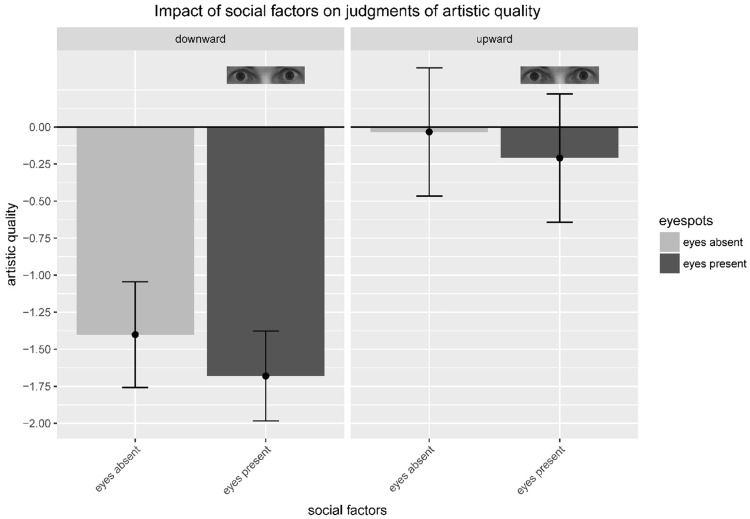
Mean ratings of artistic quality (i.e. delta between participants' ratings and baseline) split by the factors conformity pressure (downward, upward) and eyespots (absent, present). Error bars indicate 95% confidence intervals (CIs) calculated following the description of Morey (2008) for CI of means in within-subject designs.

In order to test the impact of our experimental manipulations on judgments of artistic quality, we ran a 2 × 2 within-subjects analysis of variance (ANOVA) with the factors *conformity pressure* (upward, downward) and *eyespots* (present, absent). As dependent measure, we used the delta between our participants’ judgments and the baseline rating. This is in line with the approach of Madden (1960), and further accounts for the fact that, in the present experiment, the fake ratings used for exerting conformity pressure were relative to the baseline.^1^ For each participant and trial, the delta value was calculated by the formula Δ = (participant’s judgment − baseline rating); accordingly, a positive delta value indicates that participants’ judgments were higher than the baseline, and a negative delta value indicates that participants’ judgments were lower than the baseline. Our results show significant main effects of conformity pressure, *F*(1, 47) = 72.30, *p* < .0001, η_p_^2 ^= .606, and eyespots, *F*(1, 47) = 5.09, *p* = .0288, η_p_^2 ^= .098, respectively. There was no significant interaction of the two factors, *F*(1, 47) < 1, *p* = 0.6348, *ns*. Judgments were significantly lower under downward conformity pressure (*M*_Δ_ = −1.54, *SD* = 0.88) than under upward conformity pressure (*M*_Δ_ = −0.12, *SD* = 1.16). Both the delta values were negative on a numerical basis—in the case of downward conformity pressure, that is what we had expected, in the case of upward conformity pressure, however, we had expected our participants’ judgments to be higher than the baseline. Post hoc *t*-tests revealed that the delta was significantly different from baseline only for downward conformity pressure, *t*(47) = −12.14, *p* < .0001, with a very large effect indicated by Cohen’s *d* = 1.758, but we could not reveal any significant effect for upward conformity pressure, *t*(47) = −0.72, *p* = .4734, *ns*. The descriptive data further show that the mere presence of eyespots reduced ratings of artistic quality (Figure 2).

The data assessed with the art expertise questionnaire showed that only one participant met the criteria for high expertise, thus standing out from the remainder of the sample. The latter was quite homogeneous with art expertise at low to medium levels. Recalculating the above analysis without the data of the expert participant did not lead to any changes in the reported results.

Potential modulating impacts of the assessed personality variables were tested by means of two separate multiple linear regression analyses, one for the eyespots-present setting and one for the eyespots-absent setting, respectively. As predictors, we included the following measures: the two higher-order factors of the Big Five defined by DeYoung et al. (2002), namely stability (comprising emotional stability/inverted neuroticism, agreeableness, and conscientiousness) and plasticity (comprising extraversion and openness), and the two components of social desirability according to Paulhus (2002), that is, self-deception and other-deception. All predictors were z-standardized. As dependent measure, we used the delta between judgments of artistic quality made under upward conformity pressure minus judgments made under downward conformity pressure. Neither for the eyespots-present (*R*^2 ^= .037, *p* = .8152), nor for the eyespots-absent setting (*R*^2 ^= .161, *p* = .1257) did we obtain a significant model; none of the predictors contributed significantly to explaining variance in the dependent measure.

In a last step, we analyzed the donation data, assessed at the end of the second test phase. Donations were made in the eyespots-present setting by one half of our participants, and in the eyespots-absent setting by the other half (Table 3). A two-tailed *t* test revealed that the two groups did not differ significantly with regards to the mean amount of money donated, *t*(46) = −1.69, *p* = .0984, *ns*. An undirected Mann–Whitney *U* test further showed that the groups did not differ with regard to the probability of donating money either, *z* = −1.485, *p* = .1376, *ns*.

## Discussion

Bourdieu (1968) defined art perception as a deeply sociological phenomenon. Other authors, such as Muelder Eaton (1995) and Mukařovský (2015), likewise pointed to the relevance of sociological and social factors with regards to aesthetic response and judgment. The present study is a first attempt to experimentally apply this notion to judgments of artistic quality, as opposed to mere preference ratings or impressions of beauty. We tested the impact of two specific social factors, social conformity pressure and a sense of being watched, as induced by the presence of eyespot stimuli, on judgments of artistic quality. Social conformity pressure did modulate judgments of artistic quality. This finding, however, was restricted to pressure in the downward direction, meaning our participants gave lower ratings of artistic quality for a stimulus, if would-be previous raters had unanimously judged this stimulus as being of low artistic quality. They did not give higher ratings, however, when the would-be previous raters had unanimously judged the stimulus as being of high artistic quality. Such asymmetry would be compatible with the results of Madden (1960) who tested the impact of social conformity pressure on judgments of facial beauty, and found more pronounced effects of downward as compared with upward conformity pressure. Madden argues that this asymmetry might trace back to a lowering of the participants’ equilibrium point on the scale or continuum of artistic quality, which could be caused by the introduction of a social background and an increased defensiveness in making choices, related to the more public nature of the situation. According to this rationale, downward conformity pressure, going in the same direction as the shift of the equilibrium point, would be especially effective in the presence of a social background. Note, however, that the asymmetry could also be caused by differences between the reference group and the current sample. Following Madden (1960), we used baseline ratings from an external reference group. Although this reference group stemmed from the same participant pool as the sample of the present experiment and had a comparable age-composition, it is not guaranteed that our participants would have given the same or similar baseline ratings as the reference group. This point is related to the potential subjectivity of aesthetic judgments in general (cf. statistical differentiation of private and shared taste, Hönekopp, 2006; cf. also, high proportion of private as compared with shared taste in judgments of abstract artworks, Leder, Goller, Rigotti, & Forster, 2016) and should be kept in mind as a limitation of the present results.

Another aspect to be discussed is the size of the conformity pressure effect. It is likely that for a wider range of stimulus material, the effect of conformity pressure might be smaller than the very large effect found for the present stimulus set. We had selected 20 stimuli that had received medium ratings of artistic quality in a pre-study run by Haertel and Carbon (2014). Their original stimulus set comprised 213 photographed everyday objects and contemporary art objects ranging from an ordinary cracked wine bottle to the *Lobster Telephone* created by Salvador Dalí in 1936. Our set was not only smaller, but also more homogeneous with regards to artistic quality, as we had cut the lower as well as the upper tails of the common distribution. Therefore, it may not have provided a sufficient frame of reference, which might have led our participants to seek a more informative external frame of reference, thus paying particular attention to the would-be raters’ judgments. Confronted with a more heterogeneous stimulus set that offers more of a frame of reference participants might be less prone to using such external, social information. A question more or less directly related to this point is whether the conformity pressure or social influence at work in the present experiment was rather informational or normative (cf. Deutsch & Gerard, 1955). As had been stated, a larger and more heterogeneous stimulus set would offer the participants a more realistic and representative frame of reference regarding the range of artistic quality that artworks potentially have. Even participants without extended art knowledge or expertise would thus have a more informed and less vague basis for their judgments than with the restricted and homogeneous stimulus set of the present experiment, which would also render them less dependent on using other raters’ judgments as orientation. If, in this changed context, participants’ ratings conformed less to those of the would-be raters, this would indicate that the conformity effect found in our experiment was based on informational rather than normative influence. If, however, the participants strongly conformed to the would-be raters’ judgments again, this would indicate that the found conformity effect was rather normative than informational. Still another question is whether the conformity expressed in the participants’ ratings reflects an actual change of their individual and private opinions or merely public compliance. One step to get some indication in a future study would be to reassess participants’ judgments of artistic quality for the presented stimuli after a certain delay, in the absence of conformity pressure.

Against our assumptions, the presence of eyespots did not further strengthen the conformity effect—there was no interaction of the factors: The spread between judgments made under downward versus upward conformity pressure was not increased in the eyespots-present setting. Nonetheless, the presence of eyespots significantly affected judgments of artistic quality in terms of an overall downward shift (Figure 2). Panagopoulos and van der Linden (2017) recently reported that eye-cues can induce higher levels of negative emotions such as distress and anxiety. As individuals also refer to their own emotional or affective states when assessing the value of to-be-judged objects (cf. affect-as-information, e.g., Clore & Huntsinger, 2007), a negative emotion or affect can lead to devaluation. We suppose that this might have been the path that led to the overall downward shift of artistic quality judgments when eyespots were present in our experiment. This might also explain the trend why our participants numerically donated less money in the eyespots-present than in the eyespots-absent setting. A crucial question that remains open, however, is: Why did various other studies find positive and enhancing effects of eyespots on pro-social behavior? As Panagopoulos and van der Linden suggest, negative emotions elicited or increased by the presence of eyespots may lead to pro-social behavior through triggering reputational concerns or fear of being sanctioned when violating social norms. In our experiment, in contrast, the affect-as-information effect that negativized the aesthetic judgments might have overshadowed on the participants’ donating decision and behavior. Another possibility is that our participants were less responsive to the pro-sociality triggering effect of the eyespots than were participants in other studies, for instance, due to a weak chronic public self-awareness. As Pfattheicher and Keller (2015) showed, this variable potentially moderates the effect of eyespots or watching eyes on individuals’ pro-social behavior in that individuals with weaker chronic public self-awareness are less prone to show pro-social behavior in response to the presence of watching eyes than individuals with stronger public self-awareness.

As it seems, there is more but one possible path leading from the emotions or affect elicited by the presence of eyespots to specific affective, cognitive, or behavioral outcomes. The task is now to systematically investigate whether the presence of eyespots is principally related to more negative emotions or affect, or whether different kinds of eyespots maybe induce different emotional or affective responses, and, more generally, whether (depicted) eyespots elicit responses comparable with or different from those elicited by real observers. In the present experiment, the depicted eyes looked rather frightening, and likely conveyed a negative affect or an unpleasing impression (which might have directly factored in with the judgments). In addition, it would be important to gain more insight into the factors that determine which path is taken from there to a final affective, cognitive, or behavioral manifestation.

## Conclusion

In the present experiment, we showed that social conformity pressure and a sense of being watched induced by the mere presence of eyespots have an impact on judgments of artistic quality. The results underline the importance of including social factors in aesthetics research, thus adding to Makin’s (2017) “Gestalt nightmare.” It becomes obvious that overly reductionist approaches to investigating aesthetic perception and experience will fall short, not only due to the wholeness and Gestalt character of aesthetic or artistic objects but also due to their embedding in a larger contextual and social Gestalt.

## Note

1 In addition, we ran a 2 × 2 within-subjects ANOVA with the raw judgments of artistic quality as the dependent measure. This yielded the same pattern of results with significant main effects of conformity pressure, *F*(1, 47) = 79.67, *p* < .0001, η_p_^2 ^= .630, and eyespots, *F*(1, 47) = 5.65, *p* = .0216, η_p_^2 ^= .107, respectively. There was no interaction effect of the two factors. Judgments of artistic quality were again lower in the case of downward conformity pressure (*M*_no eyes_ = 4.13, *SD* = 1.07, *M*_eyes_ = 3.84, *SD* = 0.90) than in the case of upward conformity pressure (*M*_no eyes_ = 5.50, *SD* = 1.25, *M*_eyes_ = 5.31, *SD* = 1.29).
